# Supplementary material for: Controlling Trophoblast Cell Fusion in the Human Placenta—Transcriptional Regulation of Suppressyn, an Endogenous Inhibitor of Syncytin-1
Source: Biomolecules. 2023 Nov 7;13(11):1627. doi: 10.3390/biom13111627 (PMC10668956; doi:10.3390/biom13111627)
Supplement: Supplementary file 1 [file biomolecules-13-01627-s001.zip › Supplementary Figures, Table /Supplementary Figures, Table revise.pdf]

Supplementary Figure S1

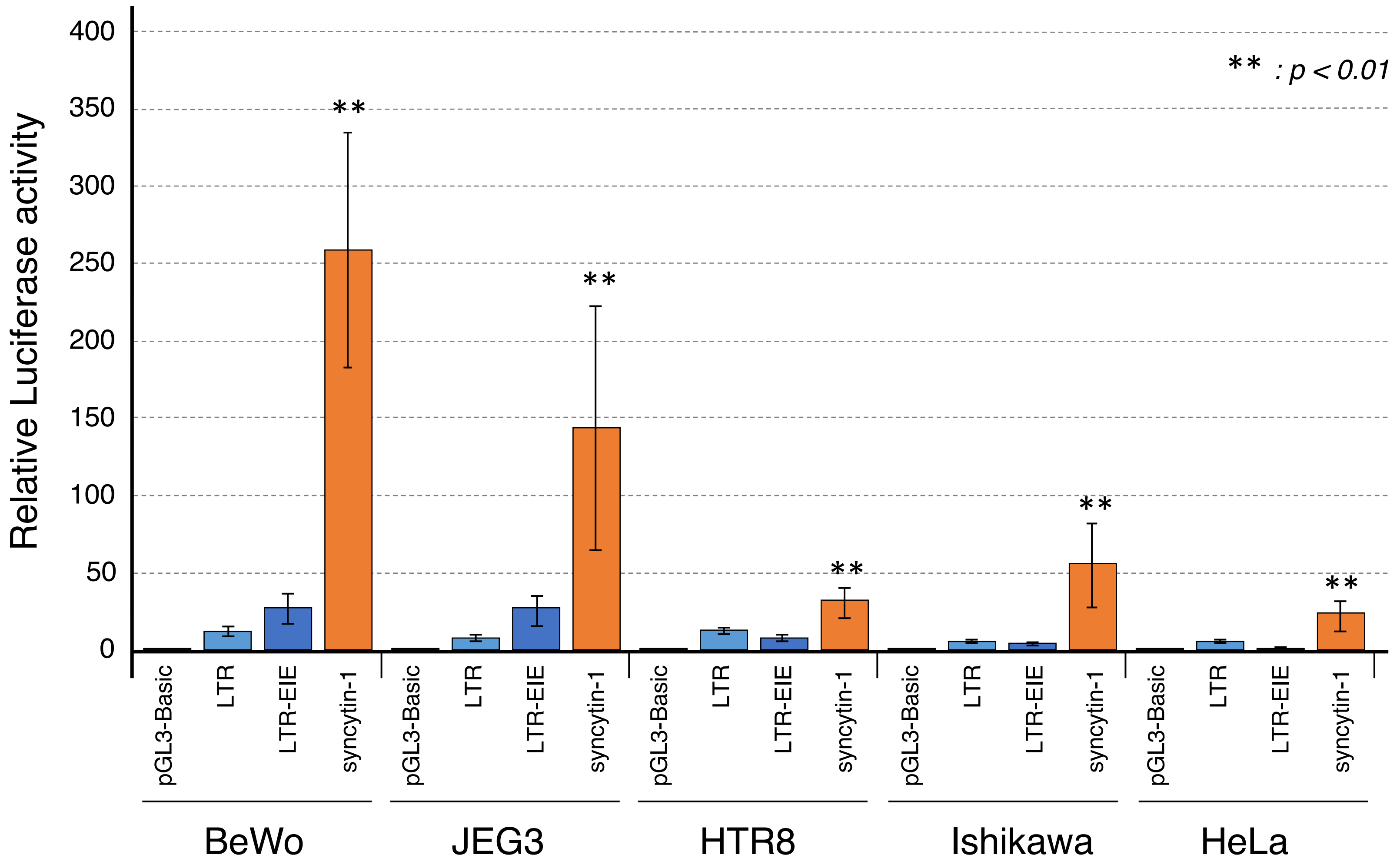

Supplementary Figure S2

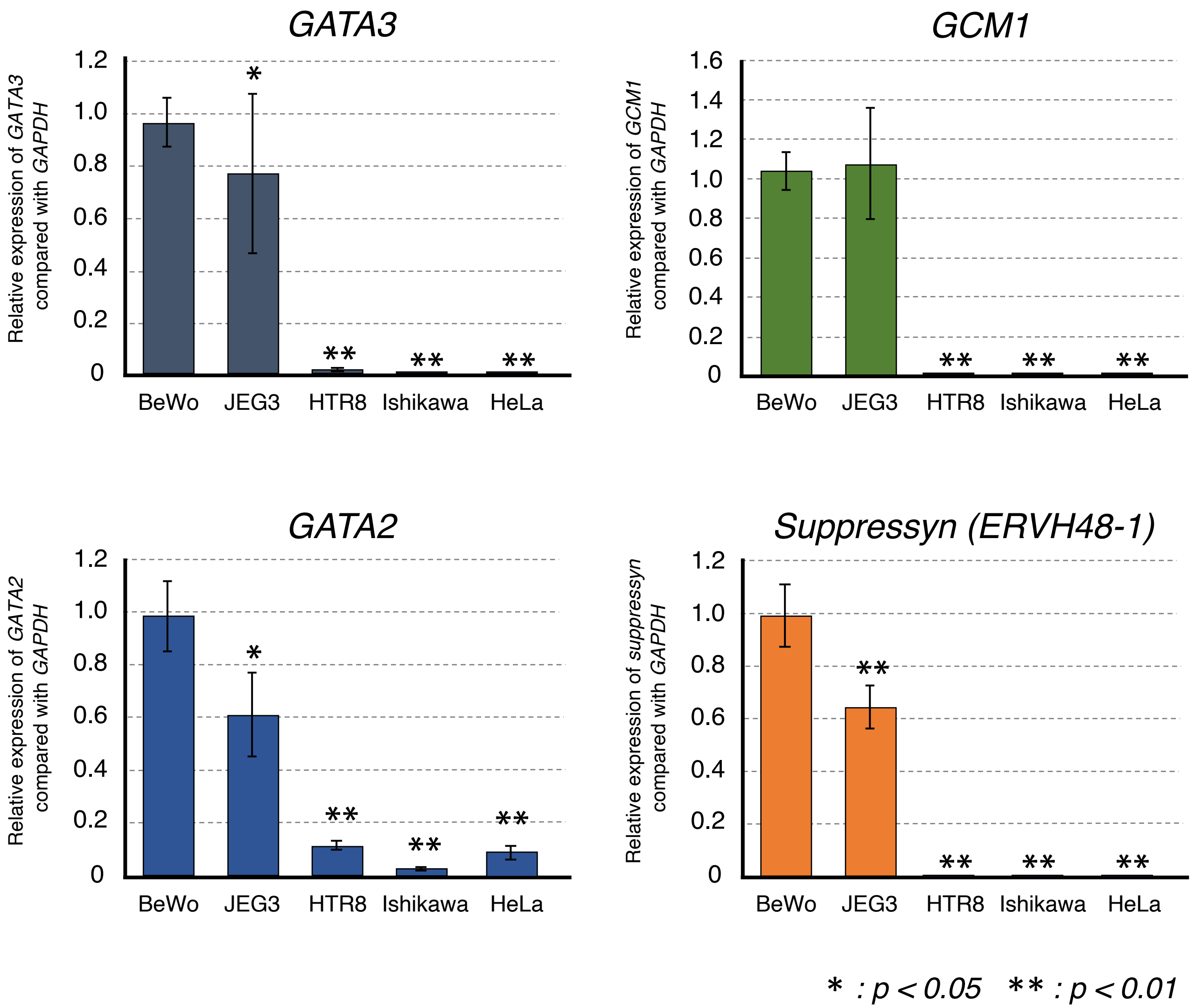

Supplementary Figure S3

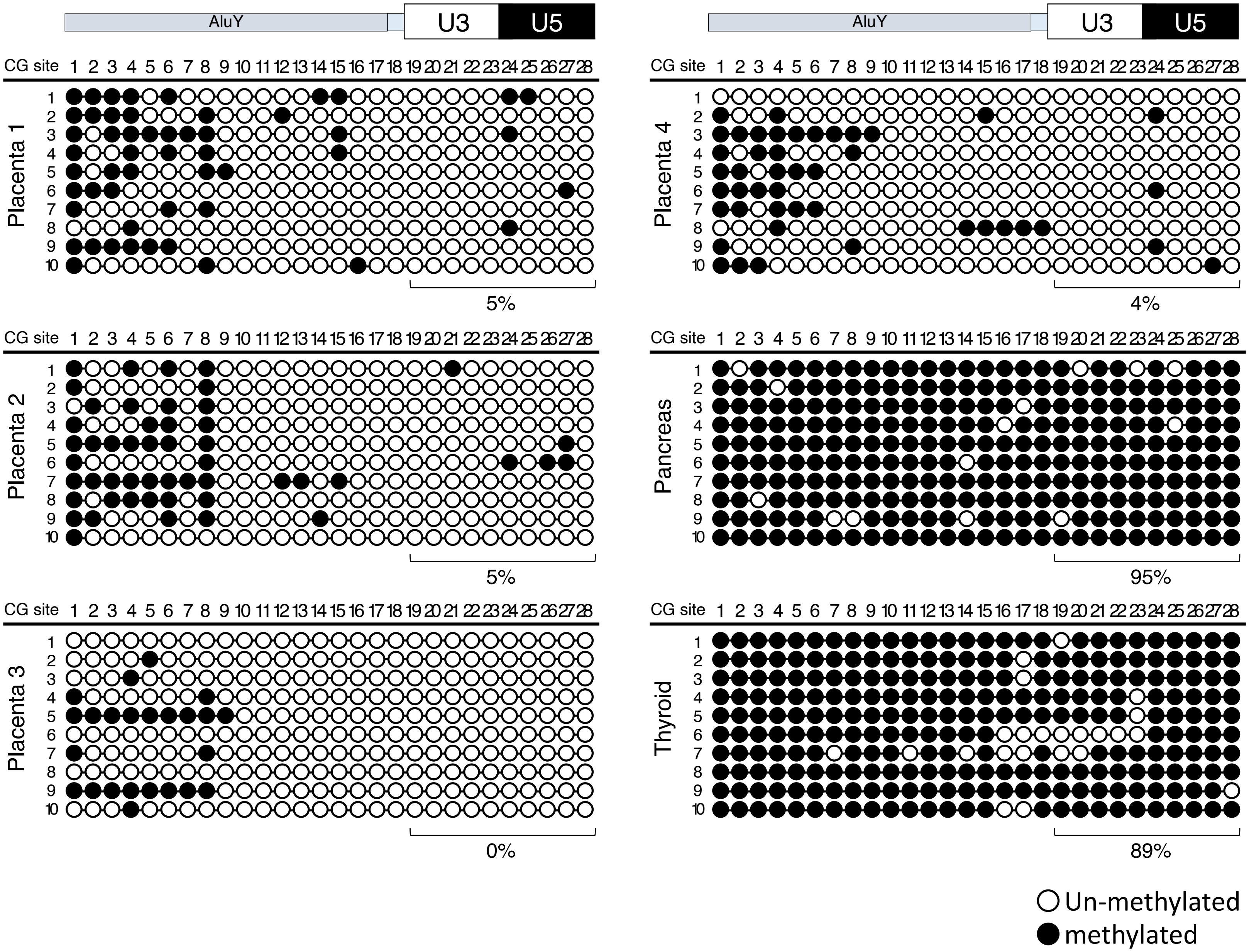

Supplementary Figure S4

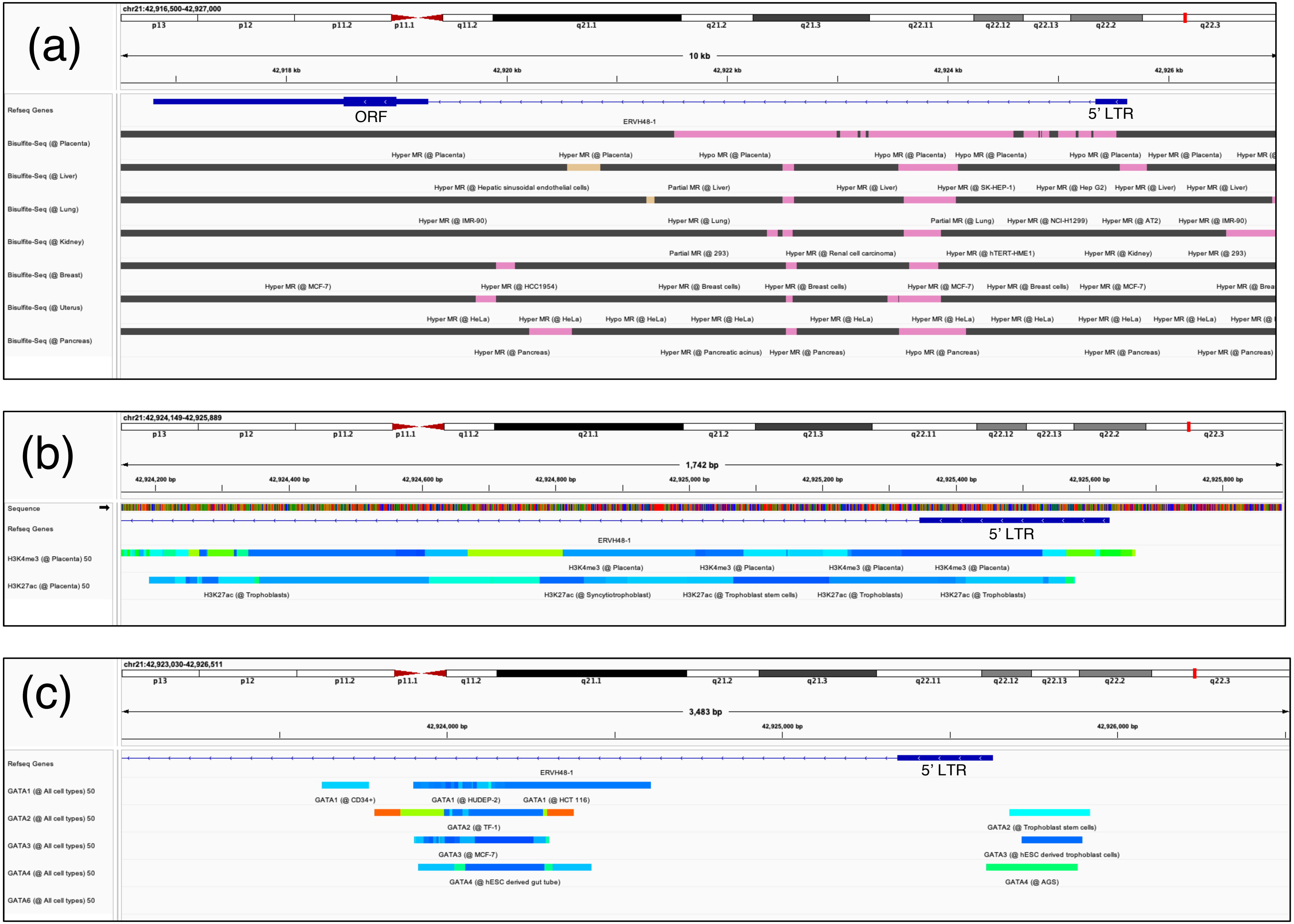

Supplementary Figure S5

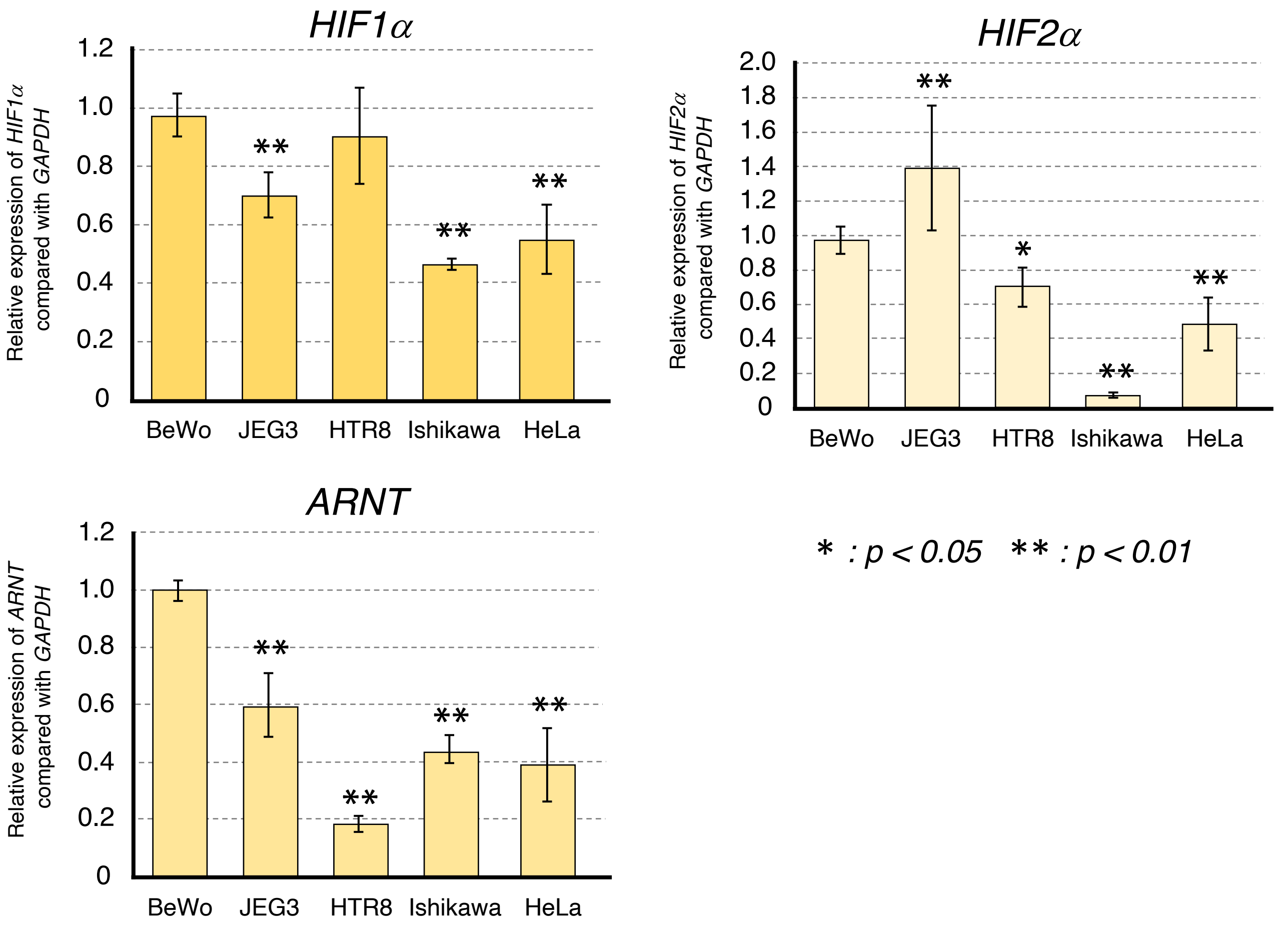

Supplementary Figure S6

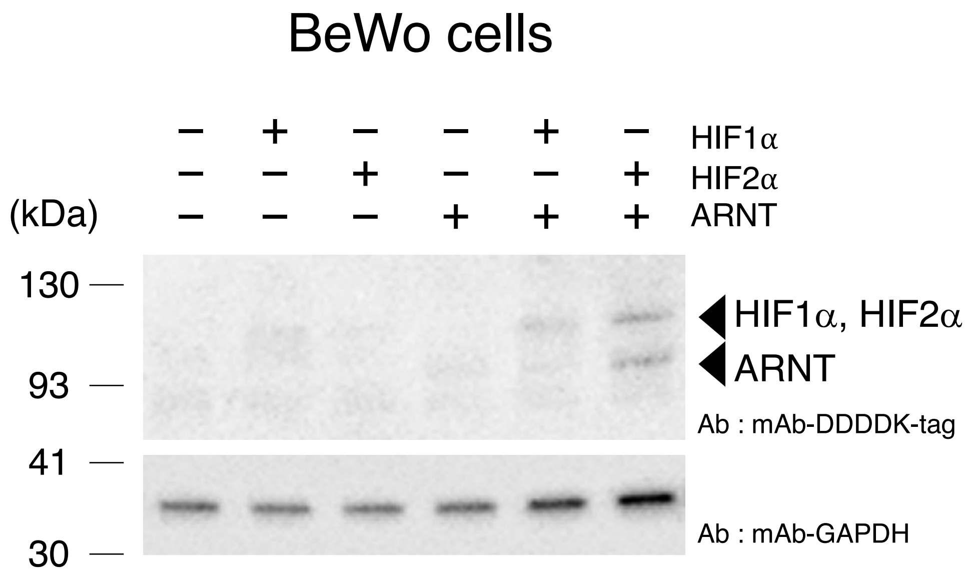

Supplementary Figure S7

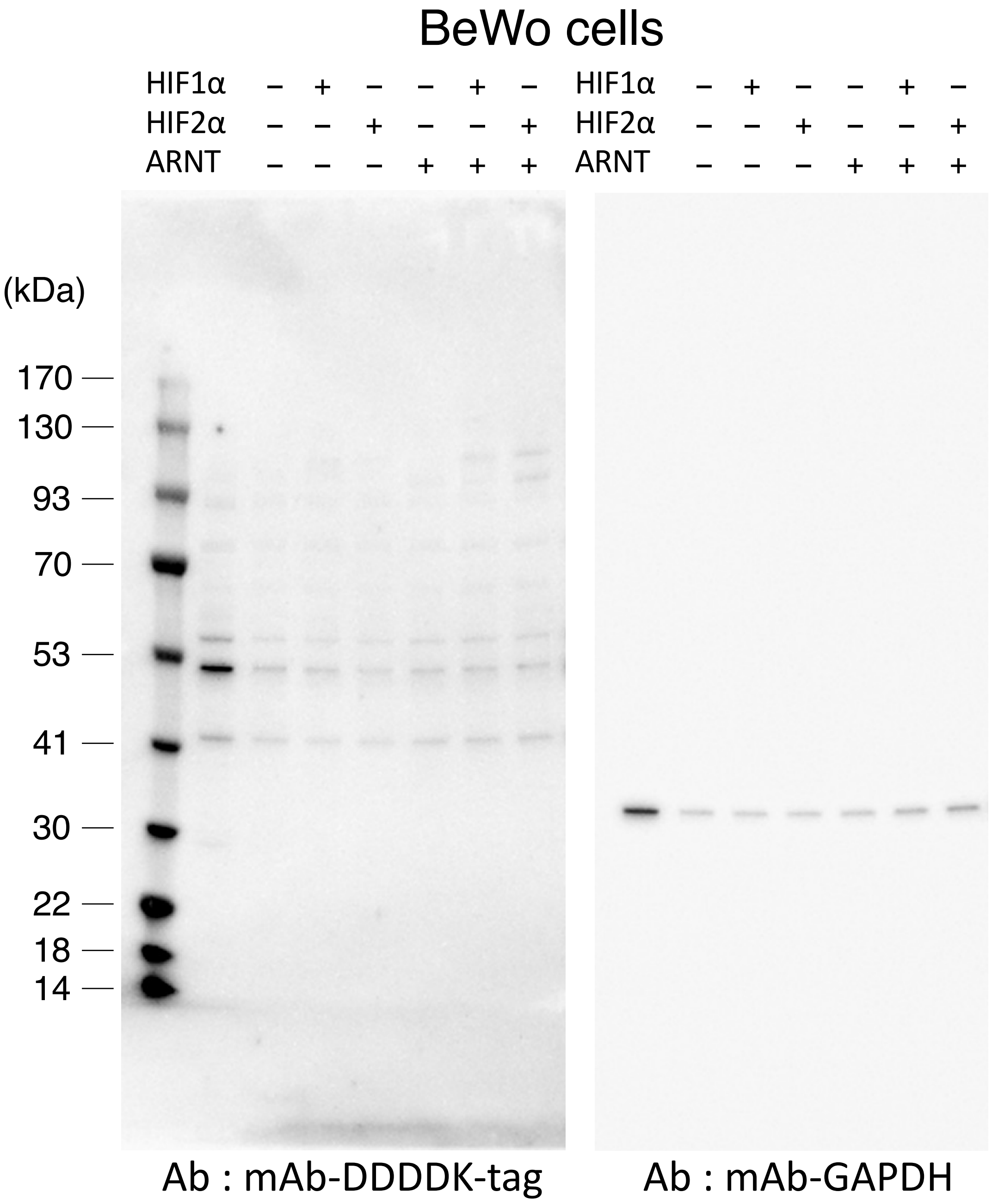

Supplementary Table S1

	Gene	Primer ID	sequence (5' - 3')
qPCR	*suppressyn (ERVH48-1)*	SUPYN-S	GAAGCTCCTCAGCCTCTAGT
		SUPYN-AS	GGGTCAGTTTGGCCGATAAA
	ARNT	ARNT-S	GGAATGGACTTGGCTCTGTAA
		ARNT-AS	GTCATCATCTGGGAGGGAAAC
	GCM1	GCM-S	AGAAGCTGAGGCAAGAAGAG
		GCM-AS	GACTTTGTGTTTCACCTGGAAG
	HIF1α	HIF1a-S	CAGACGATCATGCAGCTACTAC
		HIF1a-AS	CCCAGCAGTCTACATGCTAAAT
	HIF2α	HIF2a-S	CGCCATCATCTCTCTGGATTT
		HIF2a-AS	GCTGTGGCTCCTCAACTC
	GATA2	GATA2-S	CCTCTACTACAAGCTGCACAAT
		GATA2-AS	TCTTGCTCTTCTTGGACTTGTT
	GATA3	GATA3-S	GAACTGTCAGACCACCACAA
		GATA3-AS	GCCTTCCTTCTTCATAGTCAGG
Mutagenesis	*HIF*	LTR-HIFmg-S	TAGCAGTCAGGCTGGGACAATTCCT
		LTR-HIFmg-AS	CCAGCCTGACTGCTATGTAGTACGCG
Bisulfite sequencing	*suppressyn (ERVH48-1)*	BS-supyn-S	GGATGTGGAAAATTTGAGATAGG
		BS-supyn-AS	AAAACCAACCCCAAAACTCTTAC
Gene cloning	*HIF1α*	HIF1a-1st-S	ACTTGCCTTTCCTTCTCTTCTC
		HIF1a-1st-AS	GCTGTCTGTGATCCAGCATTA
		HIF1a-2nd-EcoRV-S	CGGATATCACCGATTCACCATGGAGGGCGC
		HIF1a-2nd-BamHI-AS	CGGATCCGGCCAGTTAACTTGATCCAAAG
	HIF2α	HIF2a-1st-S	GTTCCTCCCAGTCACCTTTC
		HIF2a-1st-AS	GCTGGCGTTAGAAATACCTAGT
		HIF2a-2nd-EcoRV-S	GGATATCGTGCTCGGCGTCTGAAC
		HIF2a-2nd-BamHI-AS	CGGATCCGGCCAGGTGGCCTGGTCCAG
	ARNT	ARNT-1st-S	GTGGCGCGGCGGCGGTGGCATCTGCGGCC
		ARNT-1st-AS	CACACTCTCTCTCACTTACTC
		ARNT-2nd-EcoRV-S	GGATATCGGCGGTGGCATCTGCGGCCATGG
		ARNT-2nd-BamHI-AS	CGGATCCGGGTATTCTGAAAAGGGGGGAA
Promoter cloning	*suppressyn (ERVH48-1)*	Pro-SUPYN-1st-S (EIE-1st-S)	GTGCGGATGTGGAAAATCTGAGA
		Pro-SUPYN-1st-AS (5Enh-1st-AS)	TCGCAGGTTTCAGGGCCAGCCCCA
		Alu-LTR-2nd-Kpn-S	GGGTACCGAGATGGAGGCTCGCTCTGTCGT
		MER-LTR-2nd-kpn-S	GGGTACCGAGACAGGTCTCTTGTTAATTTA
		LTR-2nd-kpn-S	GGGTACCTGAAAGATTCTCCCAGGGGCCTG
		LTR-2nd-Xho-AS	GCTCGAGAAGGCTCTTGCATTGGTTGG
		EIE-1st-AS	GCTGGGTCTAGGTTGGTATATTT
		EIE-2nd-XhoI-AS	ACGCCTCGAGGTCAGGACCTTGGGTGATTT
		5Enh-1st-S	CATTGGCTGAGAGGTGTCTT
		5Enh-2nd-KpnI-S	GGGTACCAGCCGTCCCCAGCTTGTC
	syncytin-1	Syn1-KpnI-S	AAGGTACCTATGTATTACCATAATTAGGCAGC
		Syn1-BglII-AS	AAAGATCTAATAGAGTGAAATAGCATGAAAACAGC
