# Supplementary material for: Controlling Trophoblast Cell Fusion in the Human Placenta—Transcriptional Regulation of Suppressyn, an Endogenous Inhibitor of Syncytin-1
Source: Biomolecules. 2023 Nov 7;13(11):1627. doi: 10.3390/biom13111627 (PMC10668956; doi:10.3390/biom13111627)
Supplement: Supplementary file 1 [file biomolecules-13-01627-s001.zip › Supplementary Figures, Table /Supplementary Materials.docx]

**Supplementary Figure Legends**

**Supplementary Figure S1. *ERVH48-1* (suppressyn) and *ERVW-1* (syncytin-1) promotor activities in various trophoblast and non-trophoblast cell lines.**

Luciferase activities measured after transfection of several promoter constructs into trophoblast (BeWo, JEG3, HTR8) and non-trophoblast (Ishikawa, HeLa) cell lines. pGL3-basic luciferase activities were used to normalize luciferase activities for other constructs within the same cell line. Statistical analyses were made using Mann-Whitney U-testing with comparison to pGL3-LTR luciferase activity in each of the cell lines (***p < 0.01*). Experiments were performed in duplicate and the means and ± SDs were calculated from three independent experiments.

**Supplementary Figure S2. Transcriptional analyses for trophoblast-specific transcription factors and *ERVH48-1* (suppressyn).**

*GATA2, GATA3, GCM1 and ERVH48-1* gene transcript levels in each cell line were determined using quantitative RT-PCR and normalized to *GADPH.* Relative fold change comparisons were made to BeWo cells (Mann-Whitney U-test: **p < 0.05*, ***p < 0.01*). Values represent means ± SDs, calculated from three independent experiments.

**Supplementary Figure S3. Bisulfite sequencing analysis of the suppressyn promoter regions in human tissues.**

Bisulfite sequencing of the *ERVH48-1* 5’ LTR and 5’ flanking region. White circles represent non-methylated and black circles represent methylated CG sites. The top column depicts the location of CG sequences, numbered 1 to 28 sequentially, with numbers 19-28 depicting the methylation status of CG sites within the 5' LTR region. The bottom column shows the methylation rates (%) for each 5’ LTR region.

**Supplementary Figure S4. In silico expression analysis using CHIP-Atlas web programs.**

a) Bisulfite sequence analysis data from various tissues and cell lines. The CHIP-Atlas (https://chip-atlas.org) was used to predict DNA methylation status at or near the *ERVH48-1* locus. Hyper methylated loci are displayed in black; loci with low methylation status are shown in pink. b) The CHIP-Atlas (https://chip-atlas.org) was used to predict histone modifications at or near the *ERVH48-1* 5’LTR and EIEs loci. H3K4me3 binding capacity is depicted in the top lane; H3K27ac in the bottom lane. c) Predicted GATA transcription factor binding sites.

**Supplementary Figure S5. Transcriptional analysis of hypoxia-induced transcription factors.**

*HIF1α, HIF2α and ARNT* gene transcript levels in each cell line were determined using quantitative RT-PCR and normalized to *GADPH*Relative fold change comparisons were made to BeWo cells (Mann-Whitney U-test: **p < 0.05*, ***p < 0.01*).Values represent means ± SDs, calculated from three independent experiments.

**Supplementary Figure S6. Translational analysis of hypoxia-induced transcription factor protein expression in BeWo cells.**

Western blot analyses using a monoclonal antibody against DDDDK-tag. Lane 1: Vector-only transfected, Lane 2: HIF1α transfected, Lane 3: HIF2α transfected, Lane 4: ARNT transfected, Lane 5: HIF1α and ARNT transfected, Lane 6: HIF2α and ARNT transfected BeWo cells. GAPDH was used as an internal control.

**Supplementary Figure S7. Protein expression of hypoxia-induced transcription factors ; full immunoblot for Supplementary Figure 5.**

Western blot analyses using a monoclonal antibody against DDDDK-tag. Lane 1: Vector-only transfected, Lane 2: HIF1α transfected, Lane 3: HIF2α transfected, Lane 4: ARNT transfected, Lane 5: HIF1α and ARNT transfected, Lane 6: HIF2α and ARNT transfected BeWo cells. GAPDH was used as an internal control.

**Supplementary Table S1. Primers used for gene and promoter cloning, mutagenesis, RT-PCR and bisulfite sequencing.**

**Supplementary Methods**

(Genomic DNA isolation from human tissues)

Human genomic DNA was extracted from four placentas, one thyroid sample and one pancreas sample using the Cica geneus DNA extraction reagent, (08178-96: Kanto Chemical, Tokyo, Japan) following the manufacturer’s instruction. Placental genomic DNAs were quantified using spectrometry and stored at -20℃ until use.

(RNA isolation and Reverse transcription)

After 24 hours in culture, cells were collected from individual cell lines with 300 μl of RLT buffer. A Qiagen RNeasy Plus kit (74134: Qiagen, Valencia, CA, USA) was used for total RNA isolation in accordance with the manufacturer’s instructions. 200ng of the total RNA was subjected to reverse transcription using a ReverTraAce kit (FSK-101: Takara, Shiga, Japan) at 30℃ for 10 minutes, 42℃ for 60 minutes and 95℃ for 5 minutes.

(Quantitative RT-PCR)

Six microliters of a 20-fold diluted reverse transcription product was used for real time RT-PCR with SYBR Green I in 25μl total volume (Applied Biosystems *Power* SYBR Green Master Mix: 4368577: Thermo Fisher Scientific, Waltham, MA, USA). The primers used for real-time PCR are listed in Supplementary Table S1. Relative gene expression analyses were performed using standard curve methods.

(Western immunoblotting)

Twenty micrograms of protein from transient transfected BeWo cells were lysed in RIPA buffer (50mM Tris-HCl, pH8.0, 150mM Sodium Chloride, 0.5w/v% Sodium Deoxycholate, 0.1w/v% Sodium Dodecyl Sulfate, 1.0w/v% NP-40 substitute with protease inhibitor (165-26021: Fuji Film, Osaka, Japan) and analyzed using standard PAGE and western immunoblotting. NuPAGE Bis-Tris 4-12% precast gels (NW04127: Thermo Fisher Scientific, Waltham, MA, USA) were used for gel electrophoresis according to the manufacturer’s instructions. Immunoblotting was performed by using the iBlot2 dry blotting system (IB21001, IB24002: Thermo Fisher scientific, Waltham, MA, USA) and the monoclonal anti-DDDDK-tag antibody (1/5000 dilution, M185: MBL, Tokyo, Japan) was used for detection. HRP (Horseradish peroxidase) signals were detected using a CCD imaging unit (Ez-Capture MG/ST: ATTO corporation, Tokyo, Japan).
